# Who Will Watch the Watchmen? The Ethico-political Arrangements of Algorithmic Proctoring for Academic Integrity

**DOI:** 10.1007/s42438-021-00273-1

**Published:** 2021-11-09

**Authors:** Jade Vu Henry, Martin Oliver

**Affiliations:** University College London, London, UK

**Keywords:** Care, Ethics, Online proctoring, Higher education, Algorithms, Artificial intelligence

## Abstract

Critics of artificial intelligence have suggested that the principles of fairness, accountability and transparency (FATE) have been used for ‘ethics washing’, in order to appease industrial interests. In this article, we develop this relational and context-dependent analysis, arguing that ethics should not be understood as abstract values or design decisions, but as socio-technical achievements, enacted in the practices of students, teachers and corporations. We propose that the ethics of using AI in education are political, involving the distribution of power, privilege and resources. To illustrate this, we trace the controversies that followed from an incident in which a student was misclassified as a cheat by an online proctoring platform during the Covid-19 lockdown, analysing this incident to reveal the socio-technical arrangements of academic integrity. We then show how Joan Tronto’s work on the *ethics of care* can help think about the politics of these socio-technical arrangements — that is, about historically constituted power relations and the delegation of responsibilities within these institutions. The paper concludes by setting the immediate need for restorative justice against the slower temporality of systemic failure, and inviting speculation that could create new relationships between universities, students, businesses, algorithms and the idea of academic integrity.

## Introduction

*Quis custodiet ipsos custodes?* (Juvenal, *Satires*, 1st-2nd Century).

A university student stands accused of academic dishonesty during the Covid-19 lockdown. She makes a TikTok video, describing how algorithmic proctoring technology flagged her for cheating during a remotely administered exam. In it, she laments how her professor assigned her a failing grade and reported her to school officials. Days later, we learn that her university’s appeal process was reassuringly swift. The professor apologized, the student’s grade was reinstated — and yet this 38-second clip of the sobbing student went viral, garnering over 3 million views on TikTok and 1 million views on Twitter in just 2 weeks. Even if justice prevailed, there was something in this student’s distress that continued to resonate with many other uneasy university students, and with the instructors who believed that it did not have to be this way, not during a protracted global health emergency.

In this paper, we stay with this residual moment of unease for what it might tell us about the ethics of engaging with artificial intelligence (AI) in higher education — not as an abstract matter of ethical codes or institutional processes, but as a political matter enacted in the practices of students, teachers and corporations. We ask, irrespective of whether justice was ultimately served, might there be better ways of being with algorithms in the university? Building on the feminist speculative ethics articulated by Puig de la Bellacasa (2011, 2017), we then show how Tronto’s landmark work on the *ethics of care* (Tronto 1993) can be deployed to think about the politics of these socio-technical arrangements — that is, about the historically constituted power relations and delegation of responsibilities within educational institutions. We conclude that those involved with monitoring academic integrity must attend to the temporalities of how ethics and justice are co-constituted through the entangled everyday practices of the university workplace.

## The Ethics of AI and Education: Working with FATE

There has been considerable debate about how AI might be governed to limit the harms that have been associated with their use. Larsson (2020), for example, has reviewed the role of guidelines in ensuring ethical and trustworthy AI, noting how principles such as fairness, accountability and transparency have been put forward to address ethical concerns (i.e. the FATE deliberations). These are sometimes combined with other principles, such as explicability, non-harmful use, responsibility and integrity. In different combinations, these principles form the basis for debate and research around the creation and use of AI, for example by acting as a point of reference for conferences and meetings (e.g. Friedler and Wilson 2018), and have been found to serve as an important foundation for building users’ trust (Shin 2020). Larsson goes on to observe, however, that ‘there are considerable differences in how these principles are interpreted; why they are considered important; what issue, domain or actors they relate to; and how they should be implemented’ (Larsson 2020: 442). He suggests that discussions of these principles tend to ignore questions of power and infrastructure and notes the growing concern that principles are being used as a form of ethics washing to appease industrial interests. As Greene et al. (2019) observe, corporations may concede the need for ‘better building’, but the idea that AI might be opposed, refused or simply not created on ethical grounds seems to be ‘off the table’.

In the realm of educational technology, researchers have begun to evaluate the ethics of online proctoring systems, making reference to FATE principles (e.g. Coghlan et al. 2021). However these ‘in principle’ assessments of AI in education arguably take corporate accounts of their services at face value, glossing over notable power asymmetries amongst the stakeholders who engage with such ethical principles (cf. Davies et al. 2021). In a study of remote proctoring during the Covid-19 campus shutdowns, Selwyn et al. raise questions about ‘the surrender of control to commercial providers, the hidden labour required to sustain “automated” systems and the increased vulnerabilities of “remote” studying’ (Selwyn et al. 2021: 1). Resonating with Larsson’s call to attend to the ‘societal challenges of relating to fairness, accountability, and transparency’ (Larsson 2020: 439), a small but growing body of critical research has engaged with the ethics of AI not just as an ethics of data and computing, but as an ethics of education which addresses, among other issues, ‘power relations between teachers and their students, and of particular approaches to pedagogy’ (Holmes et al. 2021: 18). In their work on AI for educational inclusion and diversity, Porayska-Pomsta and Rajendran argue that the principle of accountability should be understood as relational and context-dependent, involving ‘priorities and investments of different stakeholders, along with temporal fashions that determine who is accountable for decisions and actions to whom, with respect to what’ (Porayska-Pomsta and Rajendran 2019: 43). They suggest that a relational approach to accountability makes for a more ‘agile’ and ‘concrete’ ethical intervention — ‘an exchange and an ethically regulated, tractable and auditable compromise between different competing interests and gains of the decision-makers’ (ibid.).

Drawing from this emerging critical research on the ethics of AI in education and from feminist scholarship, this paper will therefore engage with the ethics of online proctoring as an empirical and relational practice. Rather than formulating principles of ‘ethical AI’ or determining whether platforms conform with FATE-related guidelines, we treat principles such as ‘fairness’, ‘accountability’ and ‘transparency’ as objects of research, tracing how these values circulate when sociotechnical relations are reassembled and the balance of power shifts. In doing so, we seek to open up questions about the purposes, the values and the kinds of relationships that are privileged in ‘the digital university’ (Jones 2013), inviting reflection on the plurality of sociomaterial practices that constitutes the ‘world’ of the academy, to locate ourselves within this world, and to begin to speculate about the kinds of new relations that are needed to enact a different narrative about digital technology (Ross 2017). We align our analysis with Feenberg’s long-standing call for a philosophy of technology where ethical values are analysed with and through constructivist accounts about technology design and use, so that rather than simply either accepting or rejecting artefacts, we might instead develop new insights into how technology might enact ‘such things as reskilled work, medical practices that respect the person, architectural and urban designs that create humane living spaces, computer designs that mediate new social forms’ (Feenberg 1999: 199).

## Relationality and Power: the Speculative Ethics of Care

We take as a point of departure Puig de la Bellacasa’s ‘speculative ethics’ (2011, 2017), which puts constructivist accounts of science, technology and society (STS) into play with Joan Tronto’s political theory on the ethics of care (Tronto 1993). This posthuman feminist approach was chosen for its (1) focus on technoscience, (2) attention to empirical practice and the materiality of ‘ethical doings’, (3) engagement with affective and asymmetric relationalities, (4) attunement to the co-constitution of ethics and politics and (5) commitment to generating a ‘speculative’ critique which offers possible alternatives for living a ‘good’ life. Rather than a moral stance or invocation for motherly love, ‘care’ is thus deployed as ‘an analytic or provocation’ (Puig de la Bellacasa 2017: 7) to explore how ethics and politics are co-constituted in the case of a distraught university student’s encounter with AI.

Contrary to those who have interpreted Tronto’s care ethics as normative (e.g. Pols 2015), Puig de la Bellacasa asserts that Tronto’s work offers ‘a vision of caring [that] presupposes heterogeneity as the ontological ground on which everything humans relate with exists … Its ontological import gives to care the peculiar significance of being a nonnormative necessity’ (Puig de la Bellacasa 2017: 70). Weaving Tronto’s care ethics together with constructivist accounts of technoscience, Puig de la Bellacasa’s approach has led to a lineage of feminist scholarship in science and technology studies (STS) which foregrounds the ‘ethico-political’ practices of care in technoscience in a wide range of settings (Lindén and Lydahl 2021). These ‘Critical Care’ studies of technology design and use illustrate how ethics often operate in tension with justice — how ethical aspirations to ‘do good’ are entangled in politically charged care practices that are ‘ambivalent, contextual, and relational’ (Martin et al. 2015: 631) and can be fraught with histories of sexism, racism, capitalism and colonialism (Murphy 2015).

## A Methodological Framework for Studying the Ethics and Politics of AI

Our analysis of the ethics and politics of AI builds specifically on Tronto’s idea that care is both an affective disposition and a politically charged activity linking people, artefacts and the environment into four inter-related registers of practice (Tronto 1993: 105–8):

- *Caring about*: noticing an unmet need for care, often by assuming the position of another person or group. As an act of identification, it is culturally and individually shaped and enacts an *ethical element of attentiveness*.
- *Taking care of*: taking responsibility to make certain that the needs are met. This might involve determining how to respond, requires a sense of agency and enacts the *ethical element of responsibility*.
- *Care-giving*: directly meeting the needs of care. This requires physical work, usually involves contact and enacts the *ethical element of competence*.
- *Care-receiving*: the care-receiver responds to care-giving, enabling the care-giver to observe the response and make judgments about the sufficiency and success of caring work. It enacts the *ethical element of responsiveness*.

Understanding care in terms of these four value-laden, overlapping registers of practices makes it possible to analyse how distributions of power, privilege and resources lead to inadequate care in society, raising not only questions of ‘“For whom?” but also “Who cares?” “What for?” “Why do ‘we’ care?” and mostly “How to care?”’ (Puig de la Bellacasa 2017: 61). In this way, both ethics and justice are understood as sociomaterial achievements, co-constituted through the situated interplay of four, far-flung registers of care practices that can be continually critiqued, re-imagined and thereby reassembled.

A second idea underpinning our analysis is that such registers of care are made visible through texts, strung together through citational practices which ‘draw renewed attention to how microlevel interactions … help to perpetuate, transform, or challenge wider social and institutional formations’ (Goodman et al. 2014: 460). Our methodological sensibilities borrow from studies of institutional interaction related to software development (Yates and Orlikowski 2002), mental health services (Günther et al. 2015) and air pollution debates (Solin 2004), which deploy the concept of *intertextuality* (Bakhtin 1981) as a method for social research (Bazerman 2003; Fox 1995; Fairclough 1992). These studies illustrate how actors are connected through *intertextual chains* corresponding to more-or-less stable constellations of shared social practices.

We recognise that intertextual work of this kind may suggest a focus on rhetoric or discourse; instead, however, we align our approach with Barad’s proposal that ‘discursive practices are not human-based activities but rather specific material (re)configurings of the world through which local determinations of boundaries, properties, and meanings are differentially enacted’ (Barad 2003: 828). In line with this postdigital perspective, we therefore view these texts as the material traces of ‘the changes that take place whenever algorithmic systems unfold in existing social contexts—when they are built, when they diffuse, and when they are used … that can reveal existing priorities within groups, organizations, and fields, as well as their changes over time’ (Christin 2020). We did not look for explicit reference to ‘care’ within these texts; instead of operating as a linguistic marker, we viewed care as achievements enacted through the material-discursive apparatus around the proctoring platform, and sought to identify what or who was implicated in such forms of care (students, values, the platform, market share, etc.).

The TikTok video that motivated our inquiry was posted in September 2020 and from this initial text, we traced four inter-textual chains made up of a total of 37 documents which we describe in terms of Tronto’s registers of care. As seen in Appendix, a variety of texts published between 2008 and 2021 were linked intertextually, either through direct quotation, indirect quotation or the mentioning of a person, document or statements (Bazerman 2003), forming the basis for the narrative in the next section. Institutional ethical approval for this research was secured prior to gathering data, based on the British Educational Research Association guidelines (BERA 2018); however, we were also mindful of ethical guidelines such as those of the Association of Internet Researchers (franzke et al. 2020). Specifically, we considered how to balance individuals’ rights to privacy and confidentiality with their moral right to be identified as authors, and the potential for harm. Where individuals or companies had created public texts (such as press releases, academic papers, blog posts, etc.), we refer here to authors as they are identified in those texts. We have not included direct quotes from social media and have avoided naming the student who created the initial video, or the institution in which she was studying.

## Proctoring Technology during Covid-19: an Ethico-Political Controversy

### ‘Caring About’ Exam Security — an EdTech Policy Network for Academic Integrity

*Caring about* refers to how society determines that needs exist and how they should be addressed. This is typically done by powerful individuals in the public sphere who often turn to technoscience for answers. Our narrative begins by exploring this register of care, tracing an ‘EdTech power network’ (Williamson 2019) that connected a housebound student’s tearful TikTok video (Text 1) to a US technology firm’s efforts to *care about* exam security.

The firm, ProctorU, was contracted by the student’s university to assist with its emergency ‘pivot’ to remote instruction during the Covid-19 pandemic. The Alabama-based company had worked with distance education programs for over a decade and believed it was well-positioned to help universities move courses quickly and effectively off school campuses and into students’ homes. They promoted themselves as offering:

… a full suite of online proctoring and identity management solutions for education, professional development and credentialing organizations. With patented, 24/7 live proctoring and a fully-automated platform, both backed by artificial intelligence, ProctorU offers a powerful, convenient and cost-effective alternative to traditional test centers … (Text 2)

Scott McFarland, the company CEO, reported ‘a ten-fold increase in colleges calling, asking for help’ in the early months of the Covid-19 crisis (Text 3). By its own account, the company successfully leveraged this track record to ‘provide a secure testing environment without missing a beat’, ramping up staffing and infrastructure in swift response to dramatic surges in demand (Text 2). Financial investors were enchanted. ProctorU claimed that while the online education ‘industry’ had expanded over recent years, the pandemic emergency had ‘accelerated these growth trends beyond all expectations’, conferring the firm and its affiliates ‘enormous competitive advantage’ in this ‘multi-billion-dollar market opportunity’ (Text 4).

But ProctorU’s expansion hit a snag. In December 2020, the company was one of three private firms contacted by a panel of six Democratic US Senators who were troubled by student complaints and media reports of ‘egregious situations’ involving biased test-proctoring products. The lawmakers wrote, ‘[w]e are concerned that the software has not been designed to be inclusive and mindful of all students’ needs and that proctors are not getting the training or information they need to adequately work with and oversee students’ (Text 5). The senators also expressed concern about data privacy and the safety of students who were made to install ‘intrusive’ test-taking software and disclose extensive personal information to the company. Citing the Family Educational Rights and Privacy Act (FERPA), the Higher Education Act and Title II of the Americans with Disabilities Act, the lawmakers requested a statement from each proctoring company to ‘…address alarming equity, accessibility, and privacy issues faced by students using the platforms’.

The senators questioned whether ProctorU’s products discriminated against students on the basis of race, religion, gender or disability. In its written response (Text 6), ProctorU attempted to allay concerns by downplaying the importance of its cheating detection algorithms:

We utilize software tools as a means to assist our human proctors … [S]oftware tools supplement and inform human judgment; they do not replace human judgment. A good analogy is a smoke detector. If one goes off, a human has to decide whether there is a fire or just burnt toast.

The statement stressed the active role played by humans both inside and outside the company. It highlighted, for instance, the training and responsiveness of its human proctors and declared ‘our proctors themselves are diverse and reflect the people we serve’.

The company also explained how responsibility for the remote testing environment was distributed across numerous humans both within the company and in their clients’ organizations:

… time limits, what resources are allowed or barred, whether breaks are allowed, what actions constitute misconduct or cheating that warrants further review by the testing provider or termination of an exam—are set by the test provider, the school, or the instructor administering the test. Our proctors alert institutions or testing agencies to violations of their test policies but do not decide whether any incident merits a particular consequence.

The senator spearheading the inquiry, Richard Blumenthal, remained skeptical and demanded ‘much more transparency’ from the technology firms, promising ‘I will work on every necessary fix to ensure students are protected’ (Text 7). In this vein, *caring about* students would oblige ProctorU to disclose more information — details about software codes, data security measures, and other business practices — to convince leaders that its test-proctoring system was inclusive and fair.

However, this focus on transparency — albeit an important part of discussions about AI ethics — does not engage with all that ProctorU claims to *care about*. Demands for transparency divert attention away from the ethics of what ProctorU does make visible: its business case for deploying algorithms to *care about* the academic integrity of students (Text 8). The company states that it is ‘committed to using the latest technology to protect academic integrity and maintain the highest standard of fairness for every student’ (Text 9). It uses security and surveillance metaphors to describe ‘integrity in action’: their proctoring platforms ‘deter and prevent’ and are designed for the ‘detection and documentation’ of cheating, referred to as academic ‘breaches’. Institutions are ‘armed’ through the ‘reporting’ and ‘intervention’ capabilities of the platform (Text 10). ProctorU *cares about* the academic integrity of students in this manner because, in the words of CEO Scott McFarland:

[w]hen a degree is gained though fraud, it undermines the image and the brand of the school and it’s deeply unfair to the majority of students who work hard, study hard to have their degree undermined by those who take shortcuts and cheat. (Text 3)

ProctorU stated, ‘if educators care about the academic integrity of their exams and their programs, they will take action to secure the testing environment’ to ensure equity for all students and protect the reputation of their institutions (Text 11).

### ‘Taking Care’ of Student Integrity — Ethical Culture and the Campus

Advancing its business case, ProctorU’s CEO asserted, ‘[t]he rate of confirmed cheating attempts, no question about it cheating, would blow you away … Even during this pandemic, people are taking advantage to cheat more’ (Text 3). The company cites ‘statistics from more than 50 years of empirical research’ (Text 8) indicating that over half of all US undergraduates cheat. This research was conducted by the International Center for Academic Integrity (ICAI), an association for professionals who develop and implement ethical codes of conduct in universities. It can be said that these individuals *take care* of academic integrity in the manner described by Tronto, ‘assuming some responsibility for the identified need and determining how to respond to it’ (Tronto 1993: 106).

The need *to take care* of student integrity in the US was articulated early on by Donald McCabe, a business professor at Rutgers University who was troubled by high rates of self-reported student cheating. In 1992, he helped launch the ICAI ‘to combat cheating, plagiarism, and academic dishonesty in higher education’ and support ‘the cultivation of cultures of integrity in academic communities throughout the world’ (Text 12). Affiliated with the Kenan Ethics Program at Duke University (1997–2004) and the Rutland Institute for Ethics at Clemson University (2007–2017), ICAI established a longstanding research program to investigate ‘moral development, moral education, institutional culture and their relationship to academic integrity’ (Text 13). ICAI also developed assessment tools and consultation services to support university administrators, based on a working definition of ‘academic integrity’ as the ‘commitment to five fundamental values: honesty, trust, fairness, respect, and responsibility …, plus the courage to act on them even in the face of adversity’ (Text 14).

Arguing that universities had a ‘moral obligation as educators’ to pursue such values, McCabe and colleagues had long advocated for character education programs to develop the ‘ethical decision-making capacities and behaviors of students’ (Text 15). Led by senior ‘Student Affairs’ administrators, these programs aimed to align formal and informal cultural systems in the university with the values of integrity and principles of ‘restorative justice’. This was considered preferable to punitive ‘law-and-order’ approaches where:

… an emphasis is placed on values such as obedience, the rule of law, and deterrence. Administrators and faculty control policies and procedures and go to great lengths to monitor students’ behavior and enforce rules …, sending students a strong message that cheaters will be caught and punished severely … In our view, such a law-and-order orientation will lead only to a fear-based cheating culture (rather than an aspirational culture of integrity) in which students are motivated only to avoid getting caught. (Text 15)

The ‘ethical culture’ alternative encouraged students to engage in a dialogic moral education, with the values associated with honour codes serving as a ‘touchstone’ for multi-stakeholder deliberations over student integrity matters.

ProctorU often cited the cheating statistics the ICAI compiled, but did not engage with the recommendations that emerged from those findings. Instead, their 2016 white paper suggested that character development approaches were outdated and elitist (Text 16):

Higher education was once a place of high integrity. Stringent honor codes upheld and protected the reputation of a school and the value of its degree. Students sought a college education for the sake of learning itself … But, new realities created new demands for colleges and universities and raised the stakes for students. … As motivations for a college education have changed, an environment where academic dishonesty is common has formed. Left unchecked, this could cause poor learning environments as well as reputational and financial damage.

It concluded that in the absence of time, money and people to implement comprehensive systems for ethical cultures, university administrators could still *take care of* academic integrity by (1) establishing a ‘central policy and consistent punitive measures for infractions across all departments’; (2) reminding students early and frequently about expectations related to academic honesty; (3) following a ‘stringent identity authentication process’ that included ‘Visual confirmation’, ‘Identify confirmation’, ‘Keystroke biometrics’, and ‘Facial recognition software’ and (4) ‘[c]hoos[ing] the right technology partner’ to ‘securely identify and test students’.

As universities scrambled to emergency remote instruction in May 2020, ProctorU published a blog post quoting new research released by the National College Testing Association which claimed: ‘Faculty and staff should not make the egregious mistake of believing an honor code, signed statement of integrity, verbal acceptance of syllabi expectations, or other tacitly communicated acceptance is alone enough to sway academic dishonesty in online courses’ (Text 17). A subsequent ProctorU white paper written by higher education journalists Jeff Selingo and Karin Fischer argued that the iterative, bespoke approaches to assessment used during the Covid-19 shutdown were no longer adequate:

… Colleges adopted pass-fail policies to ease the strain of the chaos that enveloped their students’ lives. Instructors allowed mid-term grades to stand for the semester, while others looked to alternative assessments — written reflections or portfolios of student work — in place of traditional exams. Elsewhere, students were reminded of university honor codes and allowed to self-police themselves while taking tests.

With remote instruction likely to be the new normal in higher education, at least temporarily, stop-gap measures for assessing students’ work are no longer sufficient; secure approaches to delivering exams are essential for institutions to ensure student success, prove their value to tuition paying parents, and demonstrate outcomes to employers and, in some cases, graduate schools. (Text 18)

Selingo and Fischer also warned that accreditors would soon require additional quality control and oversight of online educational programs.

In the same paper, the authors referred to concerns about student privacy and accessibility as ‘common myths’ which ‘have fallen along a series of familiar fault lines that often follow debates in higher education about buying outside technology solutions’. They suggested that remote proctoring was complementary to other data-driven approaches to enhancing student learning:

As the coronavirus upends students’ lives, sticking to the status-quo seems unproductive to institutions that claim to be student-centered. The tools that college leaders often label as crucial to student success efforts — predictive analytics, electronic advising, and guided pathways — are common upstream tactics for retaining students; the full power of ultimately helping students succeed comes way downstream, in figuring out what they have actually learned.

They encouraged university administrators to tailor technology solutions according to the specific needs of their institution. They noted that remote proctoring was less useful for evaluation in dance courses or for field-based study, and that ‘ideally educators say its adoption should happen as part of a broader discussion among faculty about effective assessment’. (Text 18)

### ‘Care-giving’ Through Teaching and Learning — Integrity Through Pedagogical Encounters

While institutions may have policies about academic integrity and invest in infrastructure to support it, the day-to-day labour of interacting with students who may or may not cheat commonly falls to individual instructors. In Tronto’s terms, this *care giving* involves meeting student’s needs, through work and contact. ProctorU offered advice about how its services could help. Their position was that caring for students’ integrity is a burden for instructors — one that their algorithmic technology could help reduce (Text 19):

Here are four ways ProctorU can help you set and stick to testing goals this year:

• Save Time on Non-Teaching Tasks …

• Help Your Students Succeed by Keeping them Honest …

• Add Convenience to Your and Your Students’ Lives …

• Stay in the Know About Your Students and Your Exams …

ProctorU identified two challenges. The first concerns efficiency: as workloads increase, most instructors would welcome claims that ‘[w]e can save instructors 30 + hours of exam reviewing time per semester’. The second concerned intensification. They argued technology creates an arms race between students developing new ways to cheat and the instructors who must stop them, but which the company could take on, ‘Fighting Technology with Technology’ (Text 8).

Not everyone agreed. Swauger — a university librarian and researcher who tweeted in response to the TikTok video — had countered months earlier that there is no evidence that proctoring software effectively detects or prevents cheating (Text 20). This point was also developed by Dawson (Text 21), a researcher of educational technology and academic integrity who noted the lack of peer-reviewed evidence that these platforms deliver the technological or behavioural constraints they promise.

Another prior line of argument rejects ProctorU framing of academic integrity all together. Over a decade earlier, Gallant had observed that although the trope of ‘technology … as the predominant and almost immutable force acting against institutional integrity’ could be traced back to the printing press, it ignores how knowledge, information and authorship all evolve over time (Text 22). Her historical review argued that the ensuing controversies — e.g. disputes about whether working together was productive collaboration or collusion — show how ‘academic integrity’ is historically and socially contingent, not universal.

This perspective is shared later amongst the people who responded to the TikTok video. For instance, Swauger writes:

Technology is often blamed for creating the conditions in which cheating proliferates and is then offered as the solution to the problem it created; both claims are false … Our habit of believing that technology will solve pedagogical problems is endemic to narratives produced by the ed-tech community. (Text 23)

Gray, a Coordinator of Educational Technologies at Thompson Rivers University, extended Swauger’s argument, calling for pedagogical strategies to prevent a repeat of this situation.

Students cheat, so we’re told, and it’s our job to defend against cheating. But for most of us, that messaging wasn’t combined with any kind of training about how to create assessments. So we replicate the assignments we saw, and we replicate the attitude towards students we heard, and we wonder why nothing changes. (Text 24)

Many of the Twitter responses followed this line, questioning why closed-book exams were still being used, challenging whether recall tests measure anything useful, and asking whether any ‘real world job’ excludes talking to other people or looking up information (Text 25). In their research, Gallant and others had long argued that academic integrity is not a simple matter of ‘fixing’ students by punishing them or even developing their character, but also involves environmental factors related to pedagogical practice and the institutional environment (Text 22). In a 2020 research compendium on academic integrity, Gallant suggested that treating cheating and plagiarism as environmental failures rather than individual moral deficits opens up ‘teachable moments’, allowing for outcomes that include not only feedback to students, but also to the environment and teaching practices (Text 26).

In the same compendium, Dawson explored how cybersecurity research might inform academic integrity scholarship, suggesting educators might actively encourage ethical or ‘white hat’ expert cheaters to test the limits of educational processes and tools so that lessons can be learnt at the level of both pedagogy and the digital infrastructure of universities (Text 21). Dawson also explored instructors’ experiences of using new technologies for assessment (Text 27). He and his co-authors found that they felt driven to pursue financial and time efficiencies as class sizes grew, but felt this came at the cost of educational quality. In marked contrast to ProctorU’s problematization, efficiency and outsourcing were seen as compromises and concessions, not desired outcomes.

More positively, these researchers reported that instructors knew technology might create new ways to cheat but were more interested in the novel opportunities created for students’ self-expression. Instead of trying to prevent or control cheating, they made it less worthwhile by reducing the credit attached — or more radically, incorporated ‘cheating’ as successful strategies for learning. They recognized, for example, that collaboration and online searching are legitimate activities in many spheres of life, and so were more interested by what students could achieve when given these opportunities, rather than how they performed under exam conditions.

Gray summarized these debates around instructors’ ‘care-giving’ during the Covid-19 lockdown provocatively:

We need to let this be the moment we choose to reframe our understanding of our learners. If we can’t trust students, if the adversarial relationship will always be so much more comfortable to us that a camera in a student’s bedroom is a more likely scenario than simple trust, then I ask again: what the hell are we even doing? (Text 24)

### Student Movements in the Digital University — ‘Care-Receiving’ During a Pandemic Emergency

Tronto reminds us that those in positions of power and responsibility who *care about* or *take care of* others may misjudge what is needed, and *care-givers* may lack the competence or resources to deliver *good care*. Caring well in society therefore also requires attending to the experience of care recipients. The care received by the emotional TikTok user from ProctorU and her university was inadequate. To borrow an earlier metaphor, the smoke detector set off a false alarm and the professor mistook burnt toast for a fire. This lapse in care placed the burden of proof on the student. To defend herself, she had to obtain a video of her exam session from ProctorU and schedule meetings with the dean and her professor. Although grateful that the university eventually re-instated her academic standing, the frustrated student wanted to know why the institution had not reviewed the evidence including the video of her taking the exam or her prior course work, rather than taking the system’s classification at face value (Text 28).

Across the USA, students expressed similar frustration and apprehension about how academic integrity policies had encroached into their homes via remote proctoring platforms. Less than a week prior, the Electronic Frontier Foundation (EFF), an advocacy group ‘defending civil liberties in the digital world’ (Text 29), reported that tens of thousands of students had signed petitions calling on universities to end their contracts with proctoring technology vendors. EFF urged universities to ‘take note of this level of organized activism’ (Text 30), cautioning:

[I]t’s not just privacy that's at stake. … the petitions we’ve seen raise very real privacy concerns—from biometric data collection, to the often overbroad permissions these apps require over the students’ devices, to the surveillance of students’ personal environments—these petitions make clear that proctoring apps also raise concerns about security, equity and accessibility, cost, increased stress, and bias.

The advocacy group exhorted educational administrators to cooperate with students, professors and parents to ‘make the very real concerns about privacy, equity, and bias in technology important components of school policy, instead of afterthoughts’.

This widespread student backlash was picked up by mainstream news journals. In an article titled, ‘How It Feels When Software Watches You Take Tests’, the New York Times highlighted the difficulties experienced by low-income students, stating, ‘[t]he rigidity of online proctoring has exacerbated an already difficult year, students say, further marginalizing them at the very moments they’re trying to prove themselves’ (Text 31). The Washington Post observed that test-proctoring companies had ‘sparked a nationwide school-surveillance revolt, with students staging protests and adopting creative tactics to push campus administrators to reconsider the deals’ (Text 32). The article described one college freshman’s efforts to audit the security of his school’s proctoring software and how thousands of others had mobilized their complaints on Twitter accounts with names such as ‘Procteario’ and ‘ProcterrorU’. The article also raised questions about the assumptions made about student integrity and digital technology:

Is stopping a few cheaters worth the price of treating every student like a fraud? And how important are any of these tests, really, given the extra stress on students whose lives have already been turned inside out?

Fuelled by shared grievances, as well as callous public statements and aggressive legal manoeuvring by several proctoring technology firms, this growing and distributed collective of students cultivated a virtual ‘ethical culture’ for student integrity that was less concerned with exam security than with dismantling proctoring technology that they considered faulty, racist, ableist and an invasion of privacy. As one student newspaper wryly proclaimed, ‘[e]xam-taking has become the new airport security’ (Text 33).

Although ProctorU CEO Scott McFarland expressed regret for what had happened to the young woman in the TikTok video, he nevertheless used the case as another opportunity to promote the company’s products:

We are always disappointed when anyone has a difficult time related to a test session. This is a good example of why it's important for students and schools to have a video recording that instructors can review and thus make evidence-based determinations. (Text 34)

This statement reinforced prior messages about the inevitability of surveillance technology in remote testing. As McFarland stated in an earlier interview, ‘we may not love the idea of being on camera every time we visit a bank or go to a convenience store, but no one is suggesting taking them down’ (Text 35).

On ProctorU’s website, positive reviews from students are used to justify this stance, praising the company for widening access to higher education (Text 36):

… ProctorU helps me handle life as a mom, Army wife, full time employee, and student by offering exam times when it's most convenient for me.

… Working full-time and also going to school often times makes it challenging to schedule exams, but ProctorU is extremely flexible and convenient.

Such testimonials foreground differences between students of established online education programs and those making the abrupt pivot to remote learning due to Covid-19. Whereas the former tend to have family and/or employment constraints that make individualized, ‘anytime, anywhere’ education helpful, the latter group expected to engage in extensive peer interaction as part of ‘campus life’, ostensibly including a culture of academic integrity involving students, instructors and administrators. However, ProctorU argued that as a matter of fairness, all students — online, hybrid and campus-based — should be subject to ‘objective, quantifiable standards’ of assessment and exam security (Text 37). They reiterated that students expected administrators and instructors to preserve the academic reputation and value of their university degrees by *taking care of* exam security.

## Analysing Breakdowns and Disasters: Academic Integrity in Trouble

Tronto argues that caring well requires the ethical virtues of attentiveness, responsibility, competence and responsiveness to be enacted through integrated practices of *caring about*, *taking care of*, *care-giving* and *care-receiving*, respectively (Tronto 1993). Failures of care occur when these four registers are unaligned. Our account of the ethical controversies around proctoring platforms echoes STS studies that engage with breakdowns (Bourrier and Nova 2019) and disasters (Fortun et al. 2017) to foreground the power relations of technological innovation and the precarity of such orderings (Law 1992). In our analysis, we discerned ruptures in the social production of academic integrity on two distinct temporal scales: (1) an immediate ‘acute’ crisis of care, related to the ethics of an algorithmic platform during the Covid-19 campus closures; and (2) a slower moving, ‘chronic’ crisis of care related to educational technology’s historical entanglements with the neoliberal university and concurrent efforts to address socio-economic disparities in US higher education (cf. Williamson 2019).

### Academic Integrity as an Acute Crisis of Care

The more apparent, punctual break in routine academic integrity practices pitted students and educational scholars against technology firms and the university administrators who procured their products. Within the compressed timeframe of this ‘acute’ Covid-19-related breakdown, actors across all four registers of care intervened decisively in the Covid-19 lockdown, enacting a highly visible public controversy over whether an algorithmic platform was an ethical replacement for on-campus practices of academic integrity. ProctorU, university administrators, and some instructors swiftly deployed the system to maintain instructional continuity and protect students against ‘others’ who cheat. For ProctorU, *caring about* academic integrity in the immediate term entailed *attentiveness* to matters of security, the reputation of its academic clients and the growth of the company. Universities *took care* of academic integrity by rapidly devolving *responsibilities* for online exam invigilation to ProctorU.

The proctoring platform, however, misclassified individuals as cheaters and created new educational barriers for students of colour, the disabled and individuals living in low-income households. Instead of *receiving care* in the manner envisioned by technology vendors and universities, groups of students quickly *responded* by mobilizing on social media and organized online petitions to dismantle proctoring platforms, raising concerns related to fairness and privacy. Doubts were raised about whether the proctoring technology enhanced the *competence* of instructors as frontline *care-givers* during campus closures. Educational scholars, advocacy groups and the press amplified student demands, catching the attention of lawmakers who also *cared about* fairness and privacy but were *attentive* mostly to the issue of industry transparency and the need to scrutinize the functionality and legality of proctoring systems.

### Academic Integrity as a Chronic Crisis of Care

The acute crisis described above is nested within a second, slower, more diffuse and ongoing disruption of academic integrity practices emerging over several decades as universities work to widen student access while contending with neoliberal agendas. In this chronic context, conflicting care practices form a different divide, with ProctorU and students of established online programs on the one hand, and education scholars and campus-based students on the other. As in the acute setting, the company promoted a technology platform to help universities respond to ‘new realities’ and ‘new demands’. But in this case, *caring about* academic integrity not only involves *attentiveness* to security, academic branding, and the financial health of the company, but also to the standardized forms of learning assessment which are better-aligned with algorithmic test proctoring.

ProctorU reported positive feedback from students of online programs who are *care receivers* seeking wider access to formal education and accredited diplomas. However, ProctorU’s approach to *caring about* academic integrity stands in marked contrast to the claim that university administrators have a ‘moral obligation as educators’ to *take care of* academic integrity and assume *responsibility* for implementing a dialogic and restorative ‘ethical culture’ in their institutions. The company’s approach was also at odds with the researchers and instructors who were *caregivers* seeking resources and pedagogical reforms to subvert neoliberal logics and develop *competence* in building teacher-student relationships, extending trust and strengthening teaching missions. Viewed in the context of a ‘slow disaster’, these competing ethical claims constituted a more fundamental controversy about academic integrity, the purpose of higher education and how quality and global reputations are achieved.

## Repairing Academic Integrity in Universities

Having foregrounded the controversies surrounding two temporally distinct but nested breakdowns in academic integrity, we ask, ‘what then must be done?’ (cf. Bharti 2021). If ‘rupturing events’ are indeed, as Guggenheim suggests, ‘inherently political … because they pose questions about who should be allowed to re-compose the world and how’ (Guggenheim 2014: 4), what do the politics of practicing an ethic of care tell us about the ethics of practicing a politic of care? What then must universities do to ‘carefully’ repair these fast and slow crises? Who should repair academic integrity in the world of the university, and how?

Our analysis confirms that we should avoid conflating the chronic temporality with *authentic* care and the acute responses with *inauthentic* care (Spier 2019). Few would argue against fixing an algorithm that is discriminatory and violates privacy, for instance. And yet, this repair can work to sustain a flawed academic integrity system, even as it brings justice to individuals. By the same token, pedagogical innovations that diversify the way learners are assessed may benefit elite students who possess alternative forms of social capital, but if reforms are incompatible with employer expectations, these may disproportionately harm marginalized students who rely on education as a vehicle to gainful employment.

Drawing from Spier’s work on inclusion in higher education (Spier 2019), we argue that ‘careful’ repair of broken academic integrity systems requires ‘mixed modes’ of response that are attuned to the temporal dynamics of care practices:

This sensibility of time challenges the idea that the educators’ wisdom relies on ‘knowing-how’ (proficiency in activating invariable-normative strategies within variable situations). Nor can wisdom be reduced to ‘knowing-that’ (proficiency in pre-reflectively following invariable-normative principles to variable situations). Instead the educator’s practical wisdom of caring is better understood as ‘knowing when’. (Spier 2019: 36).

Indeed, Michael points out the importance of recognizing how pasts, presents and futures, understood as ‘entities and occasions, discourses and practices, humans and non-humans’ (Michael 2014: 240), converge within the event of a disaster. He argues that while there may be a need to ‘slow down’ the repair of such ruptures, this deceleration can be accompanied by an ‘acceleration in the processes of asking more inventive questions, of finding better meanings, and of enabling finer responses’ (Michael 2014: 244).

## Conclusions

Do we need watchmen at all, or could we instead trust our students? If we do decide that watchmen are needed, and academic staff are unable to do this, could students watch each other, shifting the balance of power towards rather than away from them? If universities conclude that they do need to outsource this responsibility to technology, how can we ensure that it is undertaken responsibly, and in accordance with the values that universities seek to promote?

In this paper, we have argued that the ethics of AI are not only abstract or decontextualised principles, but also political and practical ‘doings’. We have described how universities (perhaps) inadvertently bought into ProctorU’s ethico-political logic when they contracted out the practices of proctoring; few realised that these new algorithmic ‘watchmen’ needed watching until people had been hurt. Might there be better ways of being with algorithms in the university? We close this paper by exploring how responses might have been different, not by invoking a new code of ‘ethical AI’, but instead by responding to Puig de la Bellacasa’s ‘speculative commitment’ (Puig de la Bellacasa 2011: 96) to reassemble current relationalities into more careful academic integrity practices.

Eighteen months into the Covid-19 pandemic, as we write this conclusion, industry executives, policymakers and economists have already released a flurry of reports which sound the clarion call to ‘re-imagine education’ with technology. Authors such as Cone et al. (2021) have observed the accelerated introduction of technology during the pandemic, linking this to the possible industrialisation of higher education. As they note, the risk related to such rallying cries is that unexamined ethical and political commitments materialise through these technologies and are then incorporated into the lives of academics and students. It would perhaps be expedient to commission these algorithmic watchmen to help manage the distributed practices of academic integrity — yet it would be foolish not to watch them in turn to ensure that they discharge this responsibility appropriately. But more importantly, rushing to ‘fix’ educational problems with technological ‘solutions’ forecloses an important opportunity for dialogue that opens up new ethical and pedagogic forms of academic practice.

Staying with one moment in the lived experience of a university student during the lockdown, we find potential avenues for living better with algorithms in the often-overlooked processes of procurement. Rather than accepting the tendency for the ethico-political assumptions of business to reshape education, we pose the question of whether academics might create different kinds of relationship with developers and service providers through inventive challenges that ‘re-imagine the business case’. As university campuses procure technologies, particularly at this moment when they re-open their doors, how has Covid-19 changed the ‘business case’ for these technologies? How has the lived experience of students, instructors and families during the online pivot challenged economic understandings of what it means to learn, and what we have lost (Strauss 2021)? As universities commission or procure technology and infrastructure, can they use this economic moment to demand business practices that will help create futures that are better educationally, or which widen access to groups that would otherwise be excluded? Can they contribute to futures that use algorithmic technology to redress rather than entrench the chronic crisis in Higher Education, opening up pedagogical possibilities for students and academics, rather than shutting them off in the name of efficiency?

The business case has been described as a rhetorical intervention that draws costs and benefits, risks and stakeholders into relationships (Maes et al. 2014). ProctorU’s case assured integrity by ‘de-risking’ assessment. Its financial costs are obvious — such as the licensing fees — but non-financial costs also exist, such as the pedagogic constraints the system requires. Variability is reduced by standardizing processes, but this requires standardizing forms of assessment, and inadvertently, how students behave and even what they look like. The justification offered rests on fears about a technologically driven ‘arms race’ of cheating.

Following Gallant and Dawson, an alternative would be to ‘de-risk’ in other ways — by valuing cheating for the ‘teachable moments’ it creates, attending to the chronic crisis by asking ‘white hat’ expert cheats to help people and processes learn and improve. Mirroring national awards for teaching, competitive ‘hacking’ could be used to test the integrity of different institutional practices. The financial costs are less clear — each proposed improvement might need its own business case — but these would be distributed between universities (for pedagogic developments) and industry (for the technologies that universities procure), and the benefits include rapid pedagogic development.

Alternatively, rather than viewing honour codes as naïve or ineffective, we could follow Gray’s provocation to trust our students, foregrounding development of integrity as a vital part of what university education is for. Instead of rewarding industry for managing educational risks, students could take this responsibility, bringing proposals for ethical action that would strengthen integrity or create better forms of assessment to their teachers, perhaps as paid work or for credit, or simply because they find such forms of co-creation more meaningful and valuable than being student-consumers (cf. Luo et al. 2019). Such engagement was visible during the acute crisis of the Covid-19 lockdown, in the student-led activism against proctoring platforms; as a response to the slow disaster, it offers sustainability and mutual development with few financial costs.

Instead of reducing risks, business cases could also explore enhanced benefits. The study by Dawson and others showed instructors adapting teaching and assessment in creative ways and being curious about what students create when cheats are shared as sensible strategies for learning, rather than deviant practices to be controlled. Rather than feeling outpaced by students in an ‘arms race’, such developments would ensure pedagogic practices remain relevant and contemporary, addressing the concerns raised earlier about employer expectations and marginalized students who need education to gain employment. This would have time costs, mainly through regular curriculum development, but the benefits should include an improved student experience, differentiation of institutions’ educational missions and which could help advance institutional ‘brand’ in a competitive student market.

ProctorU’s approach to ‘fixing’ academic integrity offers only one possible future. Alternative business cases such as these show how things could be otherwise, and what apart from risk reduction universities might value. Business cases are used to enrol stakeholders (Maes et al. 2014), so that a company like ProctorU can become the obligatory passage point for repair, moving from precarious assemblage to stable, punctualized and unquestioned ‘black boxes’. However, where business cases focus on swift repairs or immediate reparative justice, they may inadvertently sustain dysfunctional systems, exacerbating (perhaps even accelerating) the deleterious effects of slow disasters. To hold open possibilities for the careful repair of ruptures in academic integrity, this is the moment to strengthen associations with stakeholders often excluded from procurement processes, creating new relationalities between educators, technology experts, administrators, students and other-than-human actors such as algorithms that enact the values universities claim to care for: honesty, trust, fairness, respect and responsibility (Text 14). Ultimately, as with hired watchmen, a business case is only as good as the client judges it to be; it is the responsibility of university staff and students to be vigilant and engaged, so as to ensure the algorithms and platforms we chose to live with are shaped to our needs, rather than the other way around.

## Data Availability

See Appendix.

## Appendix. Text chains, by registers of care

**Table:**

Register	Text no	Title	Date created	Date retrieved	Type of text
Caring about	1	[username]	29 Sept 2020	27 Oct 2020	TikTok Video
	2	‘ProctorU Rapidly Expands Capacity to Support Campuses and Test-takers Affected by Covid-19 Concerns.' https://www.proctoru.com/industry-news-and-notes/proctoru-rapidly-expands-capacity-to-support-campuses-and-test-takers-affected-by-covid-19-concerns	23 Mar 2020	23 Jan 2021	Company press release
	3	‘A 20-Foot Cable And The Explosion Of Online Cheating.' Forbes. https://www.forbes.com/sites/dereknewton/2020/04/05/a-20-foot-cable-and-the-explosion-of-online-cheating/?sh=1a14ebba20d7	5 Apr 2020	19 Jan 2021	News article, legacy media outlet
	4	‘Gryphon Investors Completes Majority Investment in Meazure Learning.' https://www.proctoru.com/industry-news-and-notes/gryphon-investors-completes-majority-investment-in-meazure-learning	22 Dec 2020	19 Jan 2021	Company press release
	5	‘Blumenthal Leads Call for Virtual Exam Software Companies to Improve Equity, Accessibility & Privacy for Students Amid Troubling Reports.' https://www.blumenthal.senate.gov/newsroom/press/release/blumenthal-leads-call-for-virtual-exam-software-companies-to-improve-equity-accessibility-and-privacy-for-students-amid-troubling-reports	3 Dec 2020	4 Feb 2021	US government press release
	6	Prepared Written Response from ProctorU to US Senators Blumenthal (CT-D), Van Hollin (MD-D), Warren (MA-D), Wyden (OR-D), Smith (MN-D), and Booker (NJ-D). https://epic.org/privacy/dccppa/online-test-proctoring/ProctorU-senate-response-121721.pdf	17 Dec 2020	5 Feb 2021	US government testimony
	7	‘Senator: ‘More transparency is needed’ by exam proctoring tech firms.' Tech Crunch. https://techcrunch.com/2021/01/19/senator-more-transparency-is-needed-by-exam-proctoring-tech-firms/?guccounter=1	19 Jan 2021	4 Feb 2021	Online news article
	8	‘The Catch 22 of Technology in Higher Education' Educase. https://er.educause.edu/blogs/sponsored/2018/8/the-catch-22-of-technology-in-higher-education	13 Aug 2018	1 Dec 2020	Sponsored content in industry journal
	9	‘ProctorU Response to Cheating Scandal.' https://www.proctoru.com/industry-news-and-notes/proctoru-response-cheating-scandal	13 Mar 2019	1 Dec 2020	Company press release
	10	‘Integrity in Action.' https://www.proctoru.com/integrity-in-action	n.d	8 Dec2020	Company background information
	11	‘An Invitation To Cheat? New Data Unveils Student Perception and Willingness To Cheat In Unproctored Environments.' https://www.proctoru.com/industry-news-and-notes/invitation-to-cheat-new-data-unveils-student-perception-willingness-to-cheat-in-unproctored-environments	29 May 2020	1 Dec 2020	Company blog posts
Taking care of	3	‘A 20-Foot Cable And The Explosion Of Online Cheating.' Forbes. https://www.forbes.com/sites/dereknewton/2020/04/05/a-20-foot-cable-and-the-explosion-of-online-cheating/?sh=1a14ebba20d7	5 Apr 2020	19 Jan 2021	News article, legacy media outlet
	8	‘The Catch 22 of Technology in Higher Education.' Educase. https://er.educause.edu/blogs/sponsored/2018/8/the-catch-22-of-technology-in-higher-education	13 Aug 2018	1 Dec 2020	Sponsored content in industry journal
	12	Mission statement of the International Center for Academic Integrity. https://www.academicintegrity.org/about/	n.d	1 Dec 2020	Nonprofit/ advocacy org
	13	‘Our History.' International Center for Academic Integrity. https://www.academicintegrity.org/about/our-history/	n.d	9 Feb 2021	Nonprofit/ advocacy org
	14	*The Fundamental Values of Academic Integrity*. 3^rd^ Ed. International Center for Academic Integrity. https://www.academicintegrity.org/wp-content/uploads/2021/02/20019_ICAI-Fundamental-Values_R11.pdf	Feb 2021	1 Dec 2021	Nonprofit/ advocacy org
	15	*Cheating in College: Why Students Do it and What Educators Can Do about It*. Donald L. McCabe, Kenneth D. Butterfield, and Linda K. Treviño. Johns Hopkins University Press	2012	–	Academic book
	16	Cheating in the Digital Age: How Higher Education Can Protect Itself from New Forms of Academic Dishonesty. ProctorU	14 Feb 2016	–	Company white paper
	17	Academic Dishonesty and Testing: How Student Beliefs and Test Settings Impact Decisions to Cheat.(2020). Jarret M. Dyer, Heidi C. Pettyjohn, and Steve Saladin. *Journal of National College Testing,* *4*(1): 1–30. https://www.ncta-testing.org/assets/docs/JNCTA/2020%20-%20JNCTA%20-%20Academic%20Dishonesty%20and%20Testing.pdf	April 2020	18 Jan 2021	Academic Paper
	18	‘Remote Education 2.0: How Demands for Better Student Assessment are Shifting in a New Normal for Higher Education.' Jeffrey Selingo and Karin Fischer. ProctorU	2021		Company white paper
Care-giving	19	‘Four Ways ProctorU Can Help Faculty Realize Their 2020 Goals.' https://www.proctoru.com/industry-news-and-notes/four-ways-proctoru-can-help-faculty-realize-their-2020-goals	17 Feb 2020	1 Dec 2020	Company background materials
	8	‘The Catch 22 of Technology in Higher Education.' Educase. https://er.educause.edu/blogs/sponsored/2018/8/the-catch-22-of-technology-in-higher-education	13 Aug 2018	1 Dec 2020	Sponsored content in industry journal
	20	‘Software that monitors students during tests perpetuates inequality and violates their privacy.' Shea Swauger. MIT Technology Review. https://www.technologyreview.com/2020/08/07/1006132/software-algorithms-proctoring-online-tests-ai-ethics/	7 Aug 2020	5 Feb 2021	Opinion article, legacy media outlet
	21	‘Cybersecurity: the next academic integrity frontier.' Phillip Dawson. In Bretag, T. (Ed), *A Research Agenda for Academic Integrity*. Cheltenham: Edward Elgar Publishing	2020	–	Academic book
	22	‘Academic Integrity in the Twenty-First Century: A Teaching and Learning Imperative.' Trisha B. Gallant. *ASHE Higher Education Report**, 33*(5), 1–143	2008	–	Academic monograph
	23	‘Our Bodies Encoded: Algorithmic Test Proctoring in Higher Education.' Shea Swauger. Hybrid Pedagogy. https://hybridpedagogy.org/our-bodies-encoded-algorithmic-test-proctoring-in-higher-education/	2 April 2020	5 Feb 2021	Academic article
	24	‘Digital Detox #3: E-proctoring Sucks, So Why Won’t It Go Away?' Brenna Clarke Gray. Digital Detox. https://digitaldetox.trubox.ca/digital-detox-3-e-proctoring-sucks-so-why-wont-it-go-away/	2021	5 Feb 2021	Blog post
	25	[username] Reaction to TikTok video	30 Sept 2020	27 Oct 2020	Twitter Post
	26	‘Leveraging the teachable moment: what, if anything, can students learn from cheating?'” Trisha B. Gallant. In Bretag, T. (Ed), *A Research Agenda for Academic Integrity*. Cheltenham: Edward Elgar Publishing	2020	–	Academic book
	27	‘How technology shapes assessment design: Findings from a study of university teachers.' Sue Bennett, Phillip Dawson, Margaret Bearman, Elizabeth Molloy, and David Boud. *British Journal of Educational Technology**, 48* (2), 672–682	17 Feb 2017	–	Academic article
	28	‘Student Failed an Exam Because Surveillance Software Showed Her Reading the Questions out Loud.' Robin Zlotnick. Distractify. https://www.distractify.com/p/professor-failed-student-reading-out-loud	3 Oct 2020	1 Dec 2020	Online news article
Care-receiving	29	‘About EFF.' https://www.eff.org/about	n.d	1 Dec 2020	Nonprofit/ advocacy org
	30	‘Students Are Pushing Back Against Proctoring Surveillance Apps.' Jason Kelly. Electronic Frontier Foundation. https://www.eff.org/deeplinks/2020/09/students-are-pushing-back-against-proctoring-surveillance-apps	25 Sept 2020	1 Dec 2020	Nonprofit/ advocacy org
	31	‘How It Feels When Software Watches You Take Tests.' Anushka Patil and Jonah Engel Bromwich. New York Times. https://www.nytimes.com/2020/09/29/style/testing-schools-proctorio.html	29 Sept 2020	1 Dec 2020	News article, legacy media outlet
	32	‘Cheating-detection companies made millions during the pandemic. Now students are fighting back.' Drew Harwell. Washington Post. https://www.washingtonpost.com/technology/2020/11/12/test-monitoring-student-revolt/	12 Nov 2020	1 Dec 2020	News article, legacy media outlet
	33	‘You’re Being Watched: The Dangers of ProctorU.' Tara Lennon. The Review. University of Delaware. http://udreview.com/youre-being-watched-the-dangers-of-proctoru/	13 Oct 2020	9 Mar 2021	University newspaper
	34	‘A student says test proctoring AI flagged her as cheating when she read a question out loud. Others say the software could have more dire consequences.' Margot Harris. Insider. https://www.insider.com/viral-tiktok-student-fails-exam-after-ai-software-flags-cheating-2020-10	4 Oct 2020	1 Dec 2020	Online news article
	35	‘Students Are Rebelling Against Eye-Tracking Exam Surveillance Tools.' Todd Feathers and Janus Rose. Vice. https://www.vice.com/en/article/n7wxvd/students-are-rebelling-against-eye-tracking-exam-surveillance-tools	24 Sept 2020	4 Mar 2021	Online news article
	36	‘Testimonials: Don’t just take our word for it. See what our clients, partners and test-takers have to say in their own words.' https://www.proctoru.com/testimonials	n.d	20 April 2021	Company background information
	37	‘Safe And Fair Assessment Is The Key To Adult And Remote Learning.' Ashley Norris. eLearning Industry. https://elearningindustry.com/safe-fair-quality-assessment-for-adult-remote-learning	11 Jan 2021	11 Mar 2021	Sponsored content in industry journal
